# Vocabulary Learning in a Yorkshire Terrier: Slow Mapping of Spoken Words

**DOI:** 10.1371/journal.pone.0030182

**Published:** 2012-02-17

**Authors:** Ulrike Griebel, D. Kimbrough Oller

**Affiliations:** 1 Institute for Intelligent Systems, The University of Memphis, Memphis, Tennessee, United States of America; 2 School of Communication Sciences and Disorders, The University of Memphis, Memphis, Tennessee, United States of America; 3 The Konrad Lorenz Institute for Evolution and Cognition Research, Altenberg, Austria; Université Pierre et Marie Curie, France

## Abstract

Rapid vocabulary learning in children has been attributed to “fast mapping”, with new words often claimed to be learned through a single presentation. As reported in 2004 in *Science* a border collie (Rico) not only learned to identify more than 200 words, but fast mapped the new words, remembering meanings after just one presentation. Our research tests the fast mapping interpretation of the *Science* paper based on Rico's results, while extending the demonstration of large vocabulary recognition to a lap dog. We tested a Yorkshire terrier (Bailey) with the same procedures as Rico, illustrating that Bailey accurately retrieved randomly selected toys from a set of 117 on voice command of the owner. Second we tested her retrieval based on two additional voices, one male, one female, with different accents that had never been involved in her training, again showing she was capable of recognition by voice command. Third, we did both exclusion-based training of new items (toys she had never seen before with names she had never heard before) embedded in a set of known items, with subsequent retention tests designed as in the Rico experiment. After Bailey succeeded on exclusion and retention tests, a crucial evaluation of true mapping tested items previously successfully retrieved in exclusion and retention, but now pitted against each other in a two-choice task. Bailey failed on the true mapping task repeatedly, illustrating that the claim of fast mapping in Rico had not been proven, because no true mapping task had ever been conducted with him. It appears that the task called retention in the Rico study only demonstrated success in retrieval by a process of extended exclusion.

## Introduction

### Spoken word recognition in canines and children

Recently it has become clear that border collies rank with language learning animals such as great apes, parrots, and dolphins in being able to learn to understand (that is, to respond selectively to) large numbers of spoken words, gestural signals or other symbols. Kaminski, Call Fischer [1] persuasively demonstrated that Rico, an 8-year-old border collie, had learned to retrieve more than 200 items on hearing their spoken names and was able, by a process of exclusion, to chose a new item on presentation of its spoken name from a set of items for which he already knew the names. The authors further presented evidence they interpreted as “retention” of the names that had been responded to correctly in exclusion trials, and concluded that Rico had learned the names of the new items by “fast mapping”. If correct, this claim would suggest that Rico had a capability that has been characterized as a critical and truly remarkable feature of human language learning – the ability of very young children to learn semantic features of a new word on a single presentation or a very small number of presentations [2]. Fast mapping has been thought to be a primary support system for the rapid vocabulary acquisition that yields vocabularies of thousands of words in children who are still in preschool [3], [4].

Even more persuasive is the quite recent report of Chaser, a female border collie [5], who demonstrated after a massive training effort of 4–5 hours every day for three years beginning with puppyhood, that she had learned over 1000 names for objects. Pilley & Reid also responded to concerns that had been expressed about the Rico study. A critical commentary by Bloom [6] on the Rico report had pointed out that his learning of words had not been shown to apply to more than a single command type, “fetch X”, a limitation that would not occur in a normal two-year old human's understanding of words. Pilley & Reid appeared to put that concern to rest, at least for Chaser, whom they reported to have performed with perfect accuracy to commands such as *take* (pick up) X, *nose* (touch with nose) X or *paw* (touch with paw) X, where X was one of a several possible items/words in her known repertoire. Pilley & Reid also provided evidence that Chaser could learn labels for categories (i.e., common nouns), such as “toy”, which included the set of objects she was allowed to play with, in contrast to objects she was not allowed to play with, as well as two subcategories of her toys determined by shape (“Frisbees” and “Balls”). With their method, the authors were able to demonstrate that Chaser could learn three different labels for the same object at different levels of category generality (e.g. the name of a particular ball, the word “ball”, and the word “toy”). Finally, Pilley & Reid extended the demonstrations that had been made with Rico on exclusion-based selection of objects based on presentation of a new word and a new object, showing that Chaser (like Rico) was also successful in the exclusion task and on “retention” trials of the sort reported for Rico.

### A critical interpretive issue in the claim about fast mapping

The research with Rico inspired substantial excitement and commentary [6], [7], because it seemed to illustrate that dogs (at least working dogs) may have evolved through living with humans in a way that has produced a substantial capability to comprehend significant aspects of human language. The very large spoken vocabulary recognition that was demonstrated in Rico makes this point clear. The subsequent research with Chaser added considerable fuel to the fire.

However, the retention test used with Rico (and for that matter with Chaser) did not provide incontrovertible evidence of fast mapping. To understand why, consider the test sequence that was used: If a new item (call it N_x_) that had been presented as the only new item in a set was successfully retrieved by Rico based on presentation of a novel name (this was the “exclusion” task), it was later tested for “retention,” where four *known* items (call them K_1_–K_4_) were pitted against N_x_ in addition to four additional *unknown* items that had never before been presented (call them N_1_–N_4_). If Rico retrieved N_x_ on command among this set of 9, it was concluded by Kaminski et al. that he had retained the mapping of the word for N_x_ to its referent. Note, however, that success on this retention task could have resulted from Rico's merely retaining the fact that N_x_ had been presented before (or that its retrieval had been rewarded before), while K_1_–K_4_ were known items, and N_1_–N_4_ had never been presented before, nor had their retrieval been rewarded before. Thus the success could have been based on a sort of “extended exclusion” that would require retention only of items that had been presented (or rewarded) before, in addition to a differentiation of items that were known as opposed to one that had simply been presented (or rewarded) before.

Empirical support for the failure of exclusion tasks to result in fast mapping of vocabulary can even be found in literature on human children. Wilkinson et al. [8] conducted a telling study. In their “concurrent” condition, they presented two- to four- year-old children with two novel items in separate exclusion trials, where for each trial, a four-choice test set included one of the novel referents, along with three known referents (referents for which words were known). After success on this task for both novel items, children were presented with a “learning outcome” test where both of the two novel items were among the set of four, which included two known items. Fewer than half the children got the four trials that were presented in the learning outcome test correct. The authors concluded: “Children can succeed perfectly well on concurrent exposure trials … without ever remembering which novel word was paired with which novel object” (p. 756–7).

Thus, exposure to novel word-referent pairings in an exclusion test does not necessarily result in mapping of the word to the referent, even in human children. We reason that Rico, the border collie, could have succeeded on the retention test by a method of extended exclusion, where only one feature is added to the usual exclusion-based method of choice: the dog must remember items that have been presented before (or items for which a choice has been rewarded before).

### Rationale

The present report extends the evidence on dog word learning in three ways. First we ask, are border collies or perhaps working dogs, the only breeds able to learn to identify a large repertoire of items based on spoken names? Kaminski (personal communication) has indicated that, even with training, some border collies do not succeed in learning to recognize a large vocabulary of words. As for other breeds of dogs, no indications of such word learning have been reported to our knowledge.

Our report concerns a lap dog, a female Yorkshire terrier named Bailey, who was already 12 years old when we began our study. She had learned words informally with her owner, who like the owners of Rico, was not a scientist. This intrigued us, especially since Yorkshire terriers have not been bred for work and command obedience. The speculations of Kaminski and her colleagues about word learning in Rico focused on the idea that working dogs may have been specifically selected for the ability to understand human speech and other communications. So for **Experiment 1** we evaluated word recognition in Bailey, a lap dog, in a way modeled after the tests reported for Rico and Chaser, working dogs well-known for their trainability.

Second, in neither of the prior reports was there evidence presented that the dogs could generalize their recognition of words to voices other than those of their owners/trainers. **Experiment 2** tested this possibility with Bailey.

Third, we pursued the question of exclusion learning and “fast mapping” (**Experiment 3 and 4**), which had been left incomplete in the prior studies. While both Rico and Chaser had been shown capable of success in exclusion tasks, the “retention” tasks that supposedly illustrated fast mapping according to Kaminski et al. appeared to be interpretable in a way that could have involved no mapping at all, but a sort of extended exclusion-based task success only. Pilley & Reid did not comment upon the issue of fast mapping directly, though they used similar exclusion and retention tests. For **Experiment 3** and **4** we conducted the same sort of exclusion and retention tests as used with Rico and Chaser. Additionally, in **Experiment 4**, we used a direct test for mapping as a sequel to retention tests of the sort that had been used in the prior studies. Bailey failed on these tasks, suggesting that her success in exclusion and retention testing may not have resulted in word learning at all. Thus we conducted additional training and testing to find out how long it would take for Bailey to produce evidence of genuine new word learning (demonstrably distinct from exclusion-based task success), a process that took quite some time with this 12-year-old lap dog.

## General Methods

### Ethics statement

There were no human subjects participating in the research. The research participant was a pet dog only. The owner assisted us in working with the dog, and she was the only person who assisted.

No approval from an animal ethics committee was sought, because the animal in question is a pet dog, who engaged in the same games and behavioral training (with the same reinforcement food pellets) during the studies that she had engaged in with her owner through most of her life.

The owner freely participated in these studies, has seen the manuscript and all the audio-video files, as indicated in the accompanying signed statement where she agrees to having her images included in the article and confirms further that she has been given a copy of the manuscript and that she agrees to the submission of the manuscript for publication.

### Background on the experimental subject

Our subject was a female 12-year old Yorkshire terrier, Bailey. Her owner reported that Bailey had acquired about 120 toys over the years, knew each one by name and would fetch the correct one if asked for it by name. Bailey usually received treats for this behavior but was perfectly willing to do a few trials for praise alone. When Bailey received a new toy, her owner introduced the toy to her by naming it several times and letting Bailey handle it. Then the owner would throw the new toy onto a pile of Bailey's old toys and ask her to fetch it several times.

### General experimental setup

**Figure 1:**
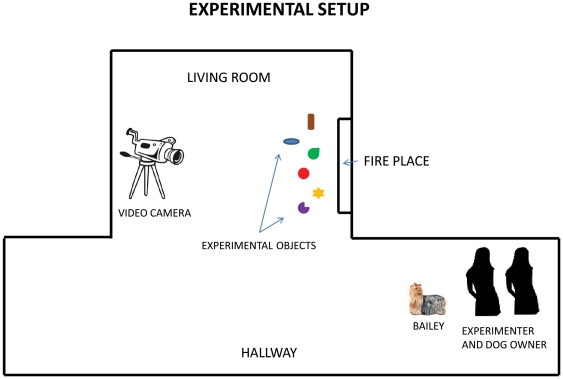
Experimental Setup. Formal trials began with the owner and the experimenter seated on the floor in the entryway out of sight from the location of the toys to be retrieved. A video camera (with no attendant) was focused on the toys, which were arrayed in front of the fireplace. After a command was given, the dog would retrieve a toy and return to the entryway. A trial was deemed correct if she had the correct toy in her mouth as soon as she came into view of the owner on return.

**Figure 1** shows the general set-up for all our experimental sessions. Since Bailey's toys were usually piled up in the living room in front of the fireplace, we chose this location for presenting the test and training trials. The primary experimenter and owner were situated around the corner from the living room in the entryway to the house, where Bailey could not see them once she turned the corner toward the fireplace to retrieve a toy. Thus there was no line of sight possible between the dog and any person during the period in which she made her choice, and in all cases the determination of whether her choice was correct was based on which toy she had in her mouth at the first instant at which she could be seen by the experimenter on returning around the corner into the entryway.

For each trial, Bailey was asked for a specific object by her owner and was sent from the entryway to the fireplace to retrieve that object and bring it to her owner (in Supporting Information see **Video S1**). After a correct choice she was rewarded with food pellets. When Bailey came back without an object, she was sent again. If this happened several times in a row, the session was either interrupted for a break or it was terminated and postponed.

All experimental sessions were videotaped and recorded with a digital video camera. Audio-video clips of the experimental sessions can be found in Supporting Information. In almost all cases the camera was on a tripod and was set to run at the beginning of the session with focus on the fireplace and the objects to be retrieved, after which the camera was not touched or adjusted until the end of the session. In addition, in order to obtain illustrative clips where the camera followed Bailey through a full trial from the location in the entryway to the location in front of the fireplace and back, a second experimenter stood with the camera and tripod for a few trials in one session, directing the camera's focus to Bailey's location and moving the camera to maintain that focus throughout the trials.

Due to Bailey's owner's work schedule, the experimental sessions were usually held on weekends, and only occasionally during the week. Thus sessions were normally one week apart, lasting 20 minutes to an hour. They were held during one of Bailey's regular feeding times and her normal food was used as positive reinforcement for the experimental trials.

There were no human subjects involved in this research. The only animal was the pet dog, whose owner participated willingly in the study.

## Experiment 1: Vocabulary recognition tests for reportedly known words Spoken by the owner

### Methods

The owner had reported that her typical way of playing with Bailey included telling her while they were in the living room to get one of the toys, which were normally in a stack of over 100 in front of the fireplace. She indicated that the dog would occasionally make mistakes, but that even in a 100-choice task, errors were rare. We had witnessed a few such informal trials earlier, on all of which Bailey had responded correctly, often having to sniff and nose through the pile to find the right object. Our experiment occurred later and addressed this apparent ability with formal experimental controls described above.

In Experiment 1we tested Bailey's recognition, based on the owner's voice command, of the names of the individual toys in her collection. To avoid any possible Clever Hans effect, the commands to retrieve one of the toys were always given by the owner from the entryway where both the owner and the experimenter were located, and the toys were always retrieved from a location out of view in front of the fireplace (see Supporting Information **Video S1**).

The primary test on vocabulary recognition for the toys was conducted in a one-hour formal session with 12 subsets of objects chosen at random from the whole set. A few of Bailey's toys could not be found on the first day of test, but 117 were available, and all formal tests on her reportedly known words were made with that set. We grouped the toys (randomly) into 11sets of 10 and one of 7 toys for Experiment 1.

To begin a test set, one of the 12 sets was arranged in front of the fireplace by the experimenter in a randomly ordered array. Two forced-choice trials were then conducted for the set. The owner called the dog to the entryway, then commanded her to retrieve one of the objects, which had been randomly determined from the group of toys in front of the fireplace. Once Bailey brought the first object, it was not put back, and she was commanded to bring a second object. Thus on the first trial, the probability was 1/10, and on the second 1/9 (or 1/7 and 1/6 in the case of the 12^th^ set). In case of error, there was no correction, but also no reward. With the 12 sets there were thus 24 trials conducted. But in addition there were many other instances in the later experiments where formal forced-choice trials were conducted with Bailey's “known” repertoire, and all these trials are relevant to the assessment of Bailey's command of the repertoire.

To be specific, in 8 subsequent sessions during Experiments 2, 3 and 4 (see below) there were 73 instances where Bailey was again commanded by her owner to retrieve objects from the reportedly known set. 29 such trials were conducted in two sessions during the Initial Exclusion Test (see Experiment 3), either as controls against novelty preference during the tests on the dog's ability to make choices based on exclusion, or as motivational trials. And subsequently, in six additional sessions where new word-training and the results of new-word training were the focus (Experiment 4, Word learning Phase I and II), 44 additional formal trials were conducted on known objects with the owner's voice, again as controls against novelty preference during exclusion tasks as well as for motivational reasons. In these 8 sessions, Bailey retrieved an object from a set ranging in size from 2 to 9 objects (M = 5.6, SD = 2.3). During the grand total of 97 trials on the dog's reportedly known set with the owner's voice, 72 of the 117 toys were tested. Due to random or semi-random selection, 49 of the 72 were tested once, 21 were tested twice and two were tested three times.

Examples of names for Bailey's toys were: Frosty the Snowman, Red Rose, Football, Nemo, Ladybug, Green Bone, Rocco Raccoon, Ozzy Ostrich, Beatrice Bat, Heidi Hippo, Iggi Iguana, Long Legged Leopard, Victor Vulture, Suzie Sunshine, Louie Lobster, Cat in the Hat, Mushy Mushroom, Twinkle Twinkle Little Star, and Rudolph the Rednosed Reindeer. Notice that Bailey's owner often chose names consisting of two or more words that included intonation and alliteration or assonance cues that may have made them easier to remember and to discriminate.

### Results on vocabulary recognition tests with the owner's voice

On 21 of the 24 trials in the first session, Bailey brought the correct toy. She missed the very first trial and then two more trials towards the end of the session. Her performance in these trials was highly statistically significant (p<0.0001). In the second and third sessions (Initial Exclusion Tests) she performed correctly on all 29 trials for known objects. For the 6 training sessions where new toys were extensively trained and some known items were tested (see below, Experiment 3), she got 42 of 44 correct on the known items. These results show extremely high levels of recognition, in all cases highly statistically significant (p<0.0001).

Out of the five total errors for the 97 recognition trials on reportedly known words with the owner's voice, three of these errors concerned a toy that was tested a single time, while the other two were tested at least one other time with positive result. 72 of the 117 toys were tested with the owner's voice, and Bailey showed overwhelming evidence of knowledge of their names, indicating that not only border collies, but, at least in one case, a lap dog is capable of learning to recognize a large vocabulary of spoken names.

## Experiment 2: vocabulary recognition with novel voices

### Methods

In this experiment we tested whether Bailey would successfully retrieve items when commanded by someone other than her owner. Experiment 2 was conducted in one session of 8 sets with two forced-choice trials for each set. Each set consisted of five of Bailey's reportedly known toys, and for each set two items were chosen randomly for Bailey to retrieve, without replacement. The total number of trials was thus 16, and the chance probability of success for each trial was 1/5 or 1/4. Due to random selection, nine of the 16 items tested had not been tested in Experiment 1 with the owner's voice.

On four of these 8 sets the first author asked Bailey for the toys (the first author is female, with a German accent), and on four sets the second author asked for the toys (the second author is male, a native American English speaker who grew up in California). Bailey's owner is very discernibly a speaker of southern American English, having grown up and lived primarily in south central and western Tennessee, and it can be said that the three speakers have extremely different voices and accents in English. Neither of the authors had ever commanded Bailey to retrieve a toy prior to the testing.

To illustrate the testing procedure in video, one of the two experimenters held the camera and moved its focus during some of these trials to track Bailey to and from the fireplace location of the objects. The other experimenter gave the voice commands from the same location in the entryway that had been used by the owner for all her test trials. The owner sat in the entryway next to the speaking experimenter during these trials (see Supporting Information **Video S2**).

### Results on vocabulary recognition with novel voices

Bailey responded quite willingly to commands produced by the novel speakers and was correct on 13 of the 16 trials. By an adaption of the binomial test this level of success was highly significant (p<0.0001). With the female voice, Bailey got 6/8 and with the male voice, 7/8; in both cases the result was significantly better than chance (p<0.0001).

Bailey succeeded with the novel voices on 7 of 9 items that had not been tested with the owner's voice. For one item, Bailey failed with the novel voice, having succeeded twice on that item with the owner's voice. For one item, she succeeded with the novel voice although she had failed with the owner's voice. The results suggested clearly that Bailey was able to recognize words in her repertoire even with novel voices.

## Experiment 3: Initial Exclusion test

Following the paradigm used with Rico, we tested whether Bailey could retrieve a toy she had never seen before from among items for which names were already known when asked for the new toy with a novel name. The choice could, of course, be made by “exclusion”, i.e. choosing the new toy from among the familiar ones on hearing a novel name.

### Methods

We conducted two sessions on two consecutive days, with 5 sets in each session (a total of 10 sets). For each set, we arranged 7 of Bailey's known toys in front of the fireplace and placed a new toy among them (a different new toy for each set). Then the owner sent Bailey from the entryway to retrieve this new toy by asking for the new toy by its novel name. To keep Bailey motivated and to provide a control against novelty preference, during each set we asked for 2–3 (randomly determined) of Bailey's known toys as well. Bailey was asked to retrieve the novel toy as the second or third item at random. Thus she could not learn to retrieve the novel item merely by its order of occurrence in the trials. The chance probability of success for the trials with the new item was thus either 1/7 or 1/6.

### Results for the Initial Exclusion Test

Bailey did not succeed in retrieval in this Initial Exclusion Test. Out of 10 sets, she only retrieved the correct item twice (once on the first day, and once on the second) when asked for the new object. This result was not statistically above chance level. During these trials, Bailey showed signs of agitation, barked often, and refused to go to retrieve the requested *new* item several times, and this occurred selectively on the trials with the new items. Reviewing the video of these sessions, we saw that in two cases Bailey handled the new toy for a while and even carried it a few steps before dropping it and picking up one of her known toys to bring to her owner. Video data were available for all of the first session but only the first seven trials of the second because of a battery failure in the camera. We examined all the trials available for these exclusion tests and found that Bailey took much longer to retrieve an incorrect object on the trials with new toys (M = 136 sec, SD = 134 sec, N = 5) than to retrieve a correct object on the trials with her known toys (all the retrievals with her known toys were correct, 29/29) (M = 15 sec, SD = 6 sec, N = 19), a difference that was statistically significant by two-tailed t-test (p<0.0004). The distributions of lag times were disjunct: i.e., none of the trials on new items where Bailey was incorrect showed a lag within the range of the lags for the known items. Interestingly, on the two trials where she correctly executed exclusion and brought the requested new toy, her response lag time was nearly on par with the trials for known items (M = 19 sec), and both trials had lags within the range of the 19 video-observed trials on known items. Thus while Bailey did not immediately adapt to retrieving new items by exclusion, she showed clear evidence of discriminating the new items from the known ones in the Initial Exclusion test.

## Experiment 4: Word Learning Test

After Bailey performed at chance level in the Initial Exclusion Test and even showed “neophobic” behavior, we decided to investigate how long it would actually take her to learn a new object name.

### Word learning Phase I

#### Methods

Since Bailey did not immediately show a pattern of exclusion performance to match that reported for Rico and Chaser, there seemed to be no point in testing immediately for retention. We reasoned that she might however be able (with a little training) to learn to overcome her apparent neophobia and retrieve new items in exclusion and retention trials. We also wanted to investigate how long Bailey would actually take to learn a new name for a new toy. So we instituted a paradigm consisting of an Informal Training (see below), followed by an exclusion trial for the new item, which was then followed by a retention trial. This sequence of Informal Training –Exclusion Trial – Retention Trial was conducted for *two* new items in each of the four sessions. To ensure that Bailey's success on the retention trial could not be based on “extended” exclusion only, we also ran a two-choice identification task at the end of each of the four sessions where we tested the two new items against each other to test whether mapping of the words to objects had occurred. We tested the same two new items until the outcome of the two-choice identification task was positive, meaning that Bailey had learned the mappings for the two new words.

The four sessions in Word learning Phase I were conducted on four different days within two weeks. In each of these sessions, tests were conducted with both novel objects (Triceratops and Dora the Explorer) sequentially. Triceratops was tested first in the first and third sessions and Dora was first in the second and fourth.

We started each session with **Informal Training** by the owner for one of the novel items, e.g., Triceratops, followed by testing for Triceratops. Then similar Informal Training was given for Dora the Explorer, followed by testing for Dora the Explorer (see Supporting Information **Video S3**). During the Informal Training, the two novel toys were introduced by Bailey's owner the way she usually introduced a new toy: She sat on the floor with Bailey in front of the fireplace, let the dog sniff and mouth the new toy, repeated the name several times, and then threw the new toy onto the pile of known toys in front of the fireplace and asked Bailey to retrieve it. Bailey always retrieved the toy, and this sequence was repeated an average of five times.

After Informal Training, three testing segments were implemented.

The first testing segment was a **Training Exclusion Test** (under controlled conditions just as in the Initial Exclusion test), in which Bailey was asked by the owner for the novel toy from among a set including 9 of her semi-randomly chosen known toys (each set throughout Training Phase I consisted of 9 different known toys) (see Supporting Information **Video S4**). As in the Initial Exclusion Test, she was asked for 1–3 of her known toys during each Training Exclusion Test to help keep her interested and to control for any possible novelty preference, and we varied the order of the trial targeting the new toy as the first, second or third item. Thus the number of items from which Dora the Explorer or Triceratops was to be chosen for each trial where one of them was requested was never less than 8 (7 “known” items plus the targeted novel item). For statistical testing we assumed conservatively, then, a chance level for response of 1/8.

The second segment was a **Retention test** (under controlled conditions) in which Bailey was presented with four known toys, four toys that she had never seen before, and the new toy (e.g., Triceratops) that had just been introduced before in Informal Training, followed by a Training Exclusion test. All the Retention tests were conducted with a completely new set of novel items except for the two targeted for learning (Triceratops and Dora the Explorer) (see Supporting Information **Video S5**). Thus with four Retention tests for Dora the Explorer and four for Triceratops along with four completely new items per test, there were a total of 32 completely new items that were assigned at random to the 8 sets. To test for statistical significance on the Retention tests we assumed the conservative chance value of 1/5 (where the denominator includes the target novel item plus the four completely new items), and ignored the fact that there were also 2 to 4 additional “known” items in each test set. None of the completely new items was ever requested.

If Bailey brought the wrong toy on any trial requesting the targeted items in the Training Exclusion Tests or the Retention Tests, she was told by the owner “no, that's not Triceratops (or Dora the Explorer)”, was given no reward, and was sent back with a second opportunity to get the novel item.

The same Retention Test design had been used with Rico, the border collie, with the intention of showing that Rico had mapped the new word to its referent, and this test had been given to him 10 minutes after the new object had been introduced in an exclusion test. With Bailey, we used an interval of 5 minutes between the Training Exclusion Test and the Retention Test. As explained above, we reasoned that Rico's success on this Retention test could have involved a sort of extended exclusion, thus providing no evidence of fast mapping. This is why we added another testing segment at the end of each session of Training Phase I: the **Two-choice Identification Test** with the two novel items as the only items placed in front of the fireplace. In each of these identification tests Bailey was asked to retrieve either Dora the Explorer or Triceratops in a total of 10 trials (five trials for each, in a random sequence).

### Results for Word Learning Phase I

**Figure 2:**
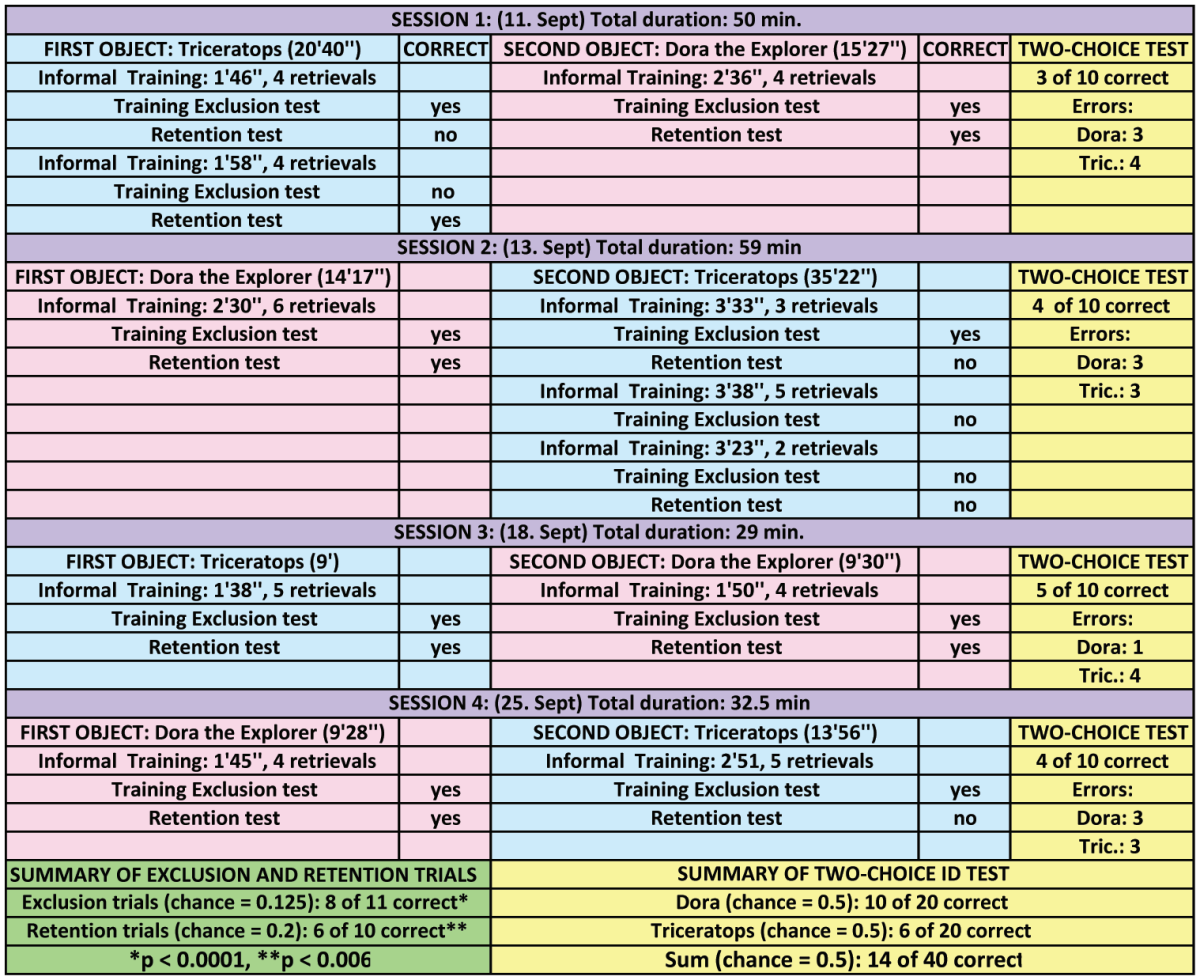
Results of Word Learning Phase I. The results in the blue and pink segments of the figure and summarized in the green segment, illustrate that Bailey was able to retrieve, at a statistically significant level, the novel objects (Dora the Explorer and Triceratops) both in the Training Exclusion and Retention tests. In the yellow segments, the data illustrate that Bailey failed utterly on the subsequent two-choice tasks that pitted the two novel items against each other. Even after four sessions, no progress had been made on the two-choice test. Before each Training Exclusion and Retention test, the owner had conducted a short set of Informal Training trials with either Dora the Explorer or Triceratops in front of the fireplace (note the time spent on the trials in minutes and seconds is recorded in the figure plus the number of retrievals for that particular Informal Training segment). Thereafter, during each session Training Exclusion and Retention tests were conducted by the primary experimenter in the formal testing setting as in Figure 1 with the novel item that had been the target during the Informal Training segment. The formal tests required two or three retrievals, one of which always targeted Dora the Explorer or Triceratops, and the others of which targeted other known items in the test set. The test set (for both Training Exclusion and Retention tests) always included known items, and in Retention also included four completely novel items that had never before been included in any test set. The columns labeled “Correct” indicate Bailey's performance *only* on the trials where Dora the Explorer or Triceratops was asked for. Bailey was virtually always correct (36 of 37 trials) when known items were requested. A two-choice task with ten trials pitting Dora the Explorer and Triceratops against each other was conducted at the end of each of the four sessions. Bailey did not exceed chance performance on the two-choice task in any of those four sessions. For additional details see text.

**Figure 2** provides a detailed account of the trials session by session as well as a summary of the results for Word Learning Phase I. The first two sessions lasted an average of 55 minutes, while the third and fourth lasted an average of 31 minutes. Bailey performed more quickly on trials in the third and fourth sessions, having seemingly gotten accustomed to the procedure. Four Training Exclusion tests and four Retention Tests were conducted for Dora the Explorer, with all trials correct (8 retrievals of Dora the Explorer and16 of known items). Because of errors with Triceratops, we conducted 3 additional (for a total of 7) Training Exclusion Tests and 2 additional (total of 6) Retention tests on Triceratops (a total of 13 retrievals where Triceratops was targeted and 21 where a known item was targeted). Bailey's relationship with Triceratops seemed to be different from that with Dora the Explorer, because she would often refuse to give Triceratops up after a retrieval and would carry the toy around as well as shake it as if in a predator-prey interaction. She never did these things with Dora the Explorer, which she simply dropped in front of her owner after each retrieval. This difference in behavior resulted in longer periods of initial informal training for Triceratops than for Dora the Explorer (mean length for Triceratops: 2′41″; mean length for Dora the Explorer: 2′10″).

Combining the data for Dora the Explorer and Triceratops, we conducted 11 Training Exclusion trials within these four sessions, and in them Bailey responded correctly 8 times (with a chance probability of 1/8, p<0.0001). She responded correctly on 6 of the 10 Retention trials (using a conservatively determined chance probability of choosing the correct novel item of 1/5, p<0.006). “Correction” trials, where Bailey went back after initially bringing a wrong object (when asked for Dora the Explorer or Triceratops), were not used for statistical analysis. At the same time it is worth noting that on 4 of the 7 errors, she did correctly retrieve the requested object (Dora the Explorer or Triceratops) on a second or third try. At the same time Bailey was nearly always correct on trials targeting a known object (36 of 37 correct).

At the end of each of these four sessions, the Two-choice Identification task with the two novel items yielded no indication that Bailey had mapped words to referents. Overall she had only 16 of 40 trials correct (for Dora the Explorer: 10 correct, 10 incorrect; for Triceratops, 6 correct, 14 incorrect). This outcome suggests that Bailey had made her choices in the Training Exclusion tests as well as in the Retention tests based on exclusion only. Clearly, success on exclusion and retention tasks of this sort (essentially the same sort used with Rico and Chaser) does not provide incontrovertible evidence of fast mapping, or for that matter of any mapping of words to referents.

### Word Learning Phase II

#### Methods

Since Bailey had not learned either of the names of the two objects (Dora the Explorer and Triceratops) after four intense sessions of exclusion-based training, we pursued a new strategy. We asked Bailey's owner to keep the targeted toys, Triceratops and Dora the Explorer, in Bailey's toy pile at home and to perform short training sessions (similar to Informal Training of Word Learning Phase I) with Bailey throughout the week, which would resemble the way Bailey would normally learn the names of new toys, in short and playful interactions. On four occasions we checked in a controlled experiment whether Bailey had in fact learned the names for the two targeted toys.

We continued testing until there was clear evidence of mapping based on two consecutive sessions with a cumulative total of correct trials significantly better than chance (which occurred by the fourth session). In the first and second of these sessions, we conducted the simple Two-choice Identification test as had been done at the end of each session in Word Learning Phase I, but in the last two sessions we used motivational trials during the test because Bailey was showing signs of experimental fatigue associated with the two new toys. This required including two to four additional objects in the choice set, all of them from the known repertoire, and also including trials requesting those known objects. Thus the set-up for the last two test sessions was different from the first two sessions, because we placed known toys together with the two new toys and intermittently had the owner request one of the known objects. These last two test sessions included altogether 10 requests for Dora the Explorer, 10 for Triceratops and 10 for a known item in random order.

### Results for Word Learning Phase II

Prior to the first two-choice identification test, the owner trained Bailey for a total of 84 minutes spread over 12 days with 66 retrievals of Dora the Explorer and 66 of Triceratops. At the end of that period, Bailey got 8 out of 12 correct trials on our formal test, but all errors occurred with Triceratops.

The owner continued informal training with an additional 5 retrievals for each item on the next day, and then we conducted a second formal test session the following day. Perhaps we tested too soon, because we had to terminate after only four trials because Bailey showed clear signs of experimental fatigue by refusing to cooperate further (one correct on each item, one incorrect on each). We gave her a few days off that were completely free of any training or testing and then continued with a light training schedule.

The owner gave only three retrieval training trials for each item during the following two weeks, and at that point we tested again, but this time with “motivational trials” asking for some of her known toys in between trials on Dora the Explorer and Triceratops. Also, for the first time in any of the testing, we allowed a large pile of Baileys known toys to be accessible during these last two sessions; they were left lying around in a pile in front of the fireplace, just behind the choice set including Dora the Explorer, Triceratops and two to four known toys which were placed in a random array in front of the pile. In this third test, Bailey got 7 of 10 trials correct.

Less than a week later, the owner trained Bailey again with four retrievals on each item. Our final formal test shortly thereafter produced 8 correct out of 10 trials on Dora the Explorer and Triceratops.

**Figure 3:**
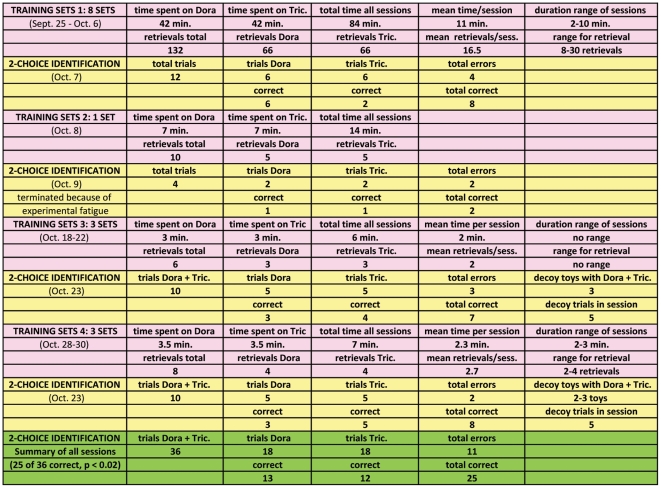
Results of Word Learning Phase II: for details see text. In Phase II, Bailey's owner spent considerable additional time training (as indicated in minutes of training and number of retrievals) on Dora the Explorer and Triceratops (without any experimenter present), and eventually the accumulated evidence from formal two-choice testing by the primary experimenter showed that Bailey had learned the mappings. The pink segments of the figure show the data on the training and the yellow segments show the results of the ten trials of two-choice testing targeting Dora the Explorer or Triceratops during each segment of testing. In green is the summary of the tests. Note that in the third and fourth segments, the test sets actually included additional known items (decoy toys) that were included to help maintain the dog's attention, and Bailey was correct each time one of those was requested. The data reported in the figure, however, only concern the trials for the target items. For additional details see text.

Thus in the final two days of testing Bailey got 15 out of 20 trials correct. On the conservative assumption of chance at 0.5, this result differs significantly from chance performance (p = 0.02). Considering all the trials conducted on the new items in Word Learning Phase II, she had 25 of 36 correct (p = 0.014). Thus it appeared that Bailey finally had learned the names for the two new toys. A detailed account on Word Learning Phase II can be found in **Figure 3**.

## Discussion

The present study indicates that extensive spoken word recognition can be learned not only by border collies, who have been artificially selected for understanding human commands for many generations and are perhaps the canine breed most well-known for general learning abilities. We have shown that a lap dog, a Yorkshire terrier, also learned to recognize a very large number of spoken words and to retrieve the corresponding items with great facility. Furthermore, the work illustrates that Bailey was capable of this kind of extensive verbal recognition even with voices and dialects other than those of her trainer/owner. Thus the research augments the groundbreaking efforts of Kaminski, Call & Fischer [1] and of Pilley & Reid [5].

It may be important to emphasize that as with Rico, the learning of this repertoire had not occurred as a result of extensive experimental training, as was conducted with Chaser. Bailey's training occurred in informal play sessions and the repertoire of items with known names grew gradually over 12 years. Maybe it played a role that Bailey had an uncommon allergy to grass and could not play outside. Consequently it seems the toy retrieval game may have become a very important feature of her life.

Our study also provides a note of caution for interpreting word learning results. The work illustrates that Rico's performance in response to novel word/object pairings, both in exclusion and retention tasks, was over-interpreted as indicating fast mapping. The performance of Bailey in tasks designed to be very similar indicates that success on such tasks can be achieved even when other evidence suggests lack of any mapping of the novel word/object pairings. Bailey succeeded in Word Learning Phase I of Experiment 4 on both exclusion tasks where two novel items were requested in the context of a set of known items and on retention tasks where the same novel items were requested in the context of several known items and several brand new ones, but then failed utterly to show successful two-choice identification retrieval of the two novel items.

This failure on the Two-choice Identification tasks in Word Learning Phase I does not prove that that Rico had failed to map the words to objects in the same circumstances; what it proves instead is that the original claim of fast mapping in Kaminski et al. [1] was not justified by the evidence. Neither success of a novel toy/word pair in an exclusion task nor success on the exclusion task plus a subsequent retention task of the sort used in these studies can by themselves prove that any kind of mapping of words to referents has been learned. There clearly remains the logical possibility that success on the tasks can be based on exclusion alone (choose the novel item) plus an extended kind of exclusion (choose the relatively novel item, the one that has been recently seen or rewarded). Some additional test of mapping must be included to prove that a word-object association has been formed.

We, like Wilkinson et al. [8], chose to test for mapping specifically by taking the precaution of training at least two items in the exclusion/retention sequence and then following with an identification task where the items that had been identified successfully in exclusion/retention were pitted against each other. In our study this was a simple two-choice task whereas in Wilkinson et al. it was a four-choice task, with the two target items paired with two known items. Wilkinson et al. termed this test the “learning outcome” task. Bailey failed on our learning outcome task, suggesting that fast mapping had not occurred.

The children in the Wilkinson et al. study *also* often failed on the learning outcome task. The exclusion/retention trials in Wilkinson et al. were composed in different ways from our work and that of Kaminski et al., for example in that the number of objects presented at the same time were different from our design. The key point here regarding Wilkinson et al.'s study is that even though the children received several “training” exclusion trials on each item and had to reach a criterion before the “learning outcome task” was given, they often failed to discriminate between the two newly “learned” items in the learning outcome task. Rico, on the other hand, was given exactly one exclusion trial with each new item, which was then tested after ten minutes in a retention trial. Success in this retention trial was taken as evidence for mapping of the name for the new item, even though there had been no testing of whether Rico could identify this new item when pitted against another item recently retrieved by exclusion. Wilkinson et al.'s study seems to call into question even for human children the idea that word learning occurs routinely by one-trial “fast mapping”. In the context of that the Wilkinson et al. finding, it seems quite a stretch to interpret Rico's result as evidence of fast mapping.

One of the reasons that Bailey showed different behaviors from Rico and Chaser in the Initial Exclusion tasks may be that by the time we tested Bailey, she was 12-years old and probably more reluctant than younger dogs to respond in novel situations. She may have been a much slower learner than she had been when she was younger. There is a reason for the saying that it is hard to teach an old dog new tricks and this is probably true for old individuals of most species capable of learning. By the time Bailey succeeded on the two-choice learning outcome task in Word Learning Phase II of experiment 4, she had been given more than 150 trials of informal retrieval experience with the two novel items in addition to considerable formal test experience with the same items over a period of more than a month. For comparison, Chaser learned in three years about 9 times as many words as Bailey had acquired. At the same time, it should be remembered that Chaser was trained 4–5 hours per day from puppyhood on spoken word recognition by experimental psychologists.

Experiments with Chaser, Rico, and Bailey place dogs among species such as chimpanzees, gorillas, orangutans, parrots, dolphins, and sea lions in their capability to understand a relatively large repertoire of human words, see e.g., [9]–[14]. Interestingly, none of the animals in these language acquisition studies were tested for the size of their *receptive* vocabulary of human words/signs since the focus of these studies was usually the *production* of human words/signs and/or other cognitive tasks. While receptive vocabulary size has not been directly tested for these animals, it seems likely (based on the reported results) that it in many cases it could be in the same range as in the dog studies that have been discussed here (a few hundred words), especially if one includes all the words that these animals appear to understand without ever being trained on them, e.g. the names of people involved in their care, household items, food items, etc. Clearly research to evaluate receptive vocabularies in other animals is in order.

All of these examples of language learning animals concern individuals who have received an enormous amount of attention and training by human caregivers. It seems possible that we might find similar abilities in other intelligent and more or less social species such as elephants, bats, pigs, ravens, mice, or rats. Given intensive human care and training, these species might perform similarly. This can only mean that many animal species have evolved some of the underlying cognitive structures required for language such as symbol and category formation [15]–[18]. The most intriguing challenge, of course, is to find out what was so special about the ancient human situation that made them take the great step further to use these capabilities extensively in both receptive and productive communication.

## Supporting Information

Video S1 Demonstration of the experimental setting. Bailey fetches Gingerbread Man (a “known” item) on owner command. In formal test trials, the owner was seated as seen in the illustration in the entryway, and could not see the dog during the choice of items, which occurred in front of the fireplace to prevent any possible visual cuing. The experimenter was also seated in the entryway during test trials (see **Figure 1**). The camera was on a tripod during formal trials with no attendant so that no one could see the dog once she disappeared from the entryway on the way to the fireplace where a set of toys was placed.
(WMV)

Video S2 An illustration of the stranger test, Experiment 2 (the second author issues the voice commands here). Target “known” toys in this illustration are Flipper and Be Mine, and both responses are correct. For both stranger voices, the dog responded correctly at much better than chance levels (p<0.0001). For the trials in this illustration, the camera was controlled by the first author, who tried to keep the focus on the dog in order to illustrate the setting fully.
(WMV)

Video S3 An illustration from Experiment 4 of Word Learning Phase I. This segment comes from the first session of Informal Training with Dora the Explorer, one of the two novel toys that were the focus of learning in Experiment 4, both Phase I and Phase II. Note that the owner conducted Informal Training with these novel items in a location in front of the fireplace, as she said she had normally done for years when a new item was being introduced to the dog for their retrieval game.
(WMV)

Video S4 From Experiment 4, Word learning Phase I, 1^st^ session, 1^st^, a Training Exclusion trial with Triceratops. The response is correct. Thereafter, there is a trial on the “known” item, Frosty the Snowman, also with a correct response. Over four such sessions with both Triceratops and Dora the Explorer as targets along with not less than 7 randomly chosen “known” items in the test set (thus conservatively a chance probability of 1/8), evidence was accumulated that Bailey could indeed perform successfully on the Exclusion test (p<0.0001) with Triceratops and Dora the Explorer.
(WMV)

Video S5 Experiment 4, Word learning Phase I, session 1, Retention test. First the known item, Stingray, is requested, and the response is correct. Then the novel item, Triceratops, is requested, and the response is incorrect. Over four such sessions with both Triceratops and Dora the Explorer as targets along with not less than 4 “completely new” items (that were never requested) in each test set (thus conservatively a chance probability of 1/5), evidence was accumulated that Bailey could indeed perform successfully on the Retention test (p<0.006) with Triceratops and Dora the Explorer. The completely new items were drawn randomly but without replacement from a set of 32 (i.e., 4 completely new items for Dora on each of 4 sets and the same for Triceratops).
(WMV)
